# Strain-level diversity of symbiont communities between individuals and populations of a bioluminescent fish

**DOI:** 10.1038/s41396-023-01550-6

**Published:** 2023-10-27

**Authors:** A. L. Gould, S. A. Donohoo, E. D. Román, E. E. Neff

**Affiliations:** 1 Institute for Biodiversity Science and Sustainability, California Academy of Sciences, SanFrancisco, CA 94121 USA; 2 School of Fisheries, Aquaculture, and Aquatic Sciences, Auburn University, Auburn, AL 36849 USA; 3 Department of Biology, Stanford University, Palo Alto, CA 94305 USA

**Keywords:** Symbiosis, Bacterial genetics

## Abstract

The bioluminescent symbiosis involving the urchin cardinalfish, *Siphamia tubifer*, and *Photobacterium mandapamensis*, a luminous member of the Vibrionaceae, is highly specific compared to other bioluminescent fish-bacteria associations. Despite this high degree of specificity, patterns of genetic diversity have been observed for the symbionts from hosts sampled over relatively small spatial scales. We characterized and compared sub-species, strain-level symbiont diversity within and between *S. tubifer* hosts sampled from the Philippines and Japan using PCR fingerprinting. We then carried out whole genome sequencing of the unique symbiont genotypes identified to characterize the genetic diversity of the symbiont community and the symbiont pangenome. We determined that an individual light organ contains six symbiont genotypes on average, but varied between 1–13. Additionally, we found that there were few genotypes shared between hosts from the same location. A phylogenetic analysis of the unique symbiont strains indicated location-specific clades, suggesting some genetic differentiation in the symbionts between host populations. We also identified symbiont genes that were variable between strains, including *luxF*, a member of the *lux* operon, which is responsible for light production. We quantified the light emission and growth rate of two strains missing *luxF* along with the other strains isolated from the same light organs and determined that strains lacking *luxF* were dimmer but grew faster than most of the other strains, suggesting a potential metabolic trade-off. This study highlights the importance of strain-level diversity in microbial associations and provides new insight into the underlying genetic architecture of intraspecific symbiont communities within a host.

## Introduction

Bacterial species are commonly characterized as being 95% similar with respect to their average nucleotide identities (ANI). In this regard, many functional or biologically relevant differences between sub-species level strains are overlooked. This is particularly true in studies of microbial symbiosis, where symbionts are often profiled by their 16 S rRNA gene identity alone. However, sub-species level profiling has become more accessible due to the advancement of genomic tools, which has allowed more studies to investigate strain-level diversity in microbial symbionts and its consequences for various hosts [1–5].

**Fig. 1:**
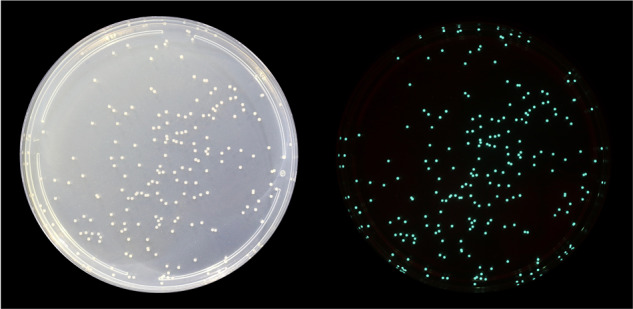
*Photobacterium mandapamensis* is the bioluminescent symbiont of *Siphamia tubifer.* These *P. mandapamensis *colonies were isolated from the light organ of *S. tubifer* and grown on LSW-70 agar plates. Photographs were taken of the same colonies in both the light (left) and dark (right).

*Photobacterium* is a genus of gram-negative bacteria in the Vibrionaceae family and is comprised of 28 described species [6], at least 7 of which are confirmed to be bioluminescent [7]. Several of the luminous *Photobacterium* species can colonize specialized tissues of fish and squid known as light organs. In particular, *P. mandapamensis* (a subspecies of *P. leiognathi* [8],) is the bioluminescent symbiont for a range of fish species, including cardinalfish in the genus *Siphamia* [9, 10] (Fig. 1). *Photobacterium mandapamensis* can also persist outside of a host’s light organ and has been isolated from coastal waters as well as from the surfaces and intestines of other marine organisms [7, 11]. Two distinct clades of *P. mandapamensis* were revealed during an analysis of the *lux* genes, which are responsible for light production, as well as the housekeeping gene *gyrB*, sequenced from the luminous symbionts of several fish hosts [10]. Most fish hosts associate with a range of *P. mandapamensis* strains in both clades as well as strains of *P. leiognathi* and a newly proposed species, *Photobacterium acropomis* sp. nov. [12]. In contrast, the sea urchin cardinalfish, *Siphamia tubifer*, appears to only associate with strains of *P. mandapamensis* in Clade II and thus, exhibits a higher degree of specificity than other fish hosts [9, 10, 12].

*Siphamia tubifer* inhabits coral reefs throughout the Indo-Pacific, sheltering in groups among the spines of sea urchins during daylight hours. At dusk, the fish leave the protection of their urchin to forage using the light emitted by its bacterial symbiont to illuminate its ventral surface [13]. While the function of this ventral luminescence has not been explicitly determined, predator avoidance by counterillumination seems most plausible. The luminous bacterial symbionts are housed in a disc-shaped light organ attached to the fish’s intestinal tract and are acquired from the environment by a larval host after at least one week of development [14, 15]. The fish can control the amount of light emitted via an opaque shutter that covers the light organ as well as the density of bacteria by regularly releasing symbiont cells into the intestine through a small duct. These bacterial cells are then shed back into the environment with fecal waste [14], a mechanism that has been hypothesized to promote local enrichment of the symbionts in the surrounding seawater and could play a role in promoting the specificity of the association [16].

Despite the high degree of specificity of the *S. tubifer*-*P. mandapamensis* symbiosis, there is strain-level symbiont diversity present within an individual host. A previous study using restriction site-associated sequencing of whole *S. tubifer* light organs indicated that an average of six symbiont types were present within a light organ; however, due to the inability to concatenate loci across the symbiont genome with that approach, this number represents a minimum estimate of the actual symbiont strain diversity [16]. The true number of symbiont strains within in a light organ as well as the diversity of strains across hosts is unknown. Furthermore, *S. tubifer* has the broadest geographic distribution of all *Siphamia* species, spanning from eastern Africa to French Polynesia [17], yet the only studies of its bioluminescent symbiosis to date have been centralized in Okinawa, Japan [10, 16]. The identity and diversity of the luminous symbionts of *S. tubifer* from other locations throughout the host’s broad Indo-Pacific range remain unknown.

In this study, we use a PCR fingerprinting approach to characterize symbiont strain diversity within *S. tubifer* light organs originating from the Philippines and compare them to those isolated from *S. tubifer*in Okinawa, Japan. Therefore, this is the first study to characterize the luminous symbionts of *S. tubifer* beyond Okinawa and provides more resolution on the amount of strain diversity present in *S. tubifer* light organs. Using the PCR fingerprints, the goal of this study is to characterize the distribution and diversity of symbiont strain types within and across hosts and populations, thus presenting new insight into the intraspecific symbiont community composition of the *S. tubifer*-*P. mandapamensis* symbiosis at these various scales. We then sequence the whole genomes of the unique symbiont genotypes to look for genetic signatures in the light organ symbionts between the host populations and characterize the symbiont pangenome. Finally, we identify and measure key phenotypic traits, luminosity and growth rate, that are variable between strains and could have meaningful consequences for the bioluminescent association with *S. tubifer*, further highlighting the importance of studying intraspecific strain-level diversity in microbial symbiosis.

## Methods and materials

### Specimen collection and bacterial isolation

*Siphamia tubifer* specimens were collected from offshore Verde Island in the Philippines (13°34’N 121°03’E) and from a site near Sesoko Island in Okinawa, Japan (26°39’N 127°52’E). Fish were euthanized following an approved IACUC protocol, and the light organs of the *S. tubifer* specimens were immediately dissected and homogenized in 0.5 mL of 1x PBS. A 10^4^ dilution of this homogenate was plated on LSW-70 (LB Lennox broth in 70% seawater) agar plates and grown for 24 h at room temperature (Fig. 1). Individual colonies were randomly selected and suspended in LSW-70 liquid media overnight at 28 °C in a shaking incubator (1600 rpms).

### PCR fingerprinting

Total DNA was extracted from cell pellets of the overnight liquid cultures for each isolate using the Qiagen DNeasy Blood and Tissue Kit spin column extraction method following the manufacturer’s instructions. For each colony, purity of the extracted DNA was measured with a Nanodrop 2000C Spectrophotometer, and the DNA concentration was measured with a Qubit dsDNA HS Assay Kit on a Qubit 2.0 Fluorometer. Extracted DNA was maintained at −20 °C until use in enterobacterial repetitive intergenic consensus-polymerase chain reactions (ERIC-PCR) [18, 19] and whole-genome sequencing.

Each ERIC-PCR was carried out in a 25 μL volume containing: 2.5 μL 10 × PCR buffer, 0.5 μL dNTPs (10 mmol/L stock), 1.0 μL MgCl_2_ (50 mmol/L stock), 0.5 μL of ERIC 1 R primer (5′-ATGTAAGCTCCTGGGGATTCAC-3′, 10 μmol/L stock), 0.5 μL of ERIC 2 primer (5′-AAGTAAGTGACTGGGGTGAGCG-3′, 10 μmol/L stock), 0.25 μL Invitrogen *Taq* (5 units/μL stock), 14.75 μL of Millepore-H_2_O and 5 μL of DNA template (50 ng, concentration 10 ng/µl). Amplification was performed using a BioRad MyCycler Thermocycler with the following protocol from Xu et al. [20]: 7 min initial denaturation at 95 °C followed by 35 cycles of 30 s denaturation at 90 °C, 1 min annealing at 52 °C, and 8 min elongated at 65 °C, with a final elongation step at 68 °C for 16 min. Amplified products were analyzed by gel electrophoresis on a 1.5% agarose gel stained with ethidium bromide. A 100-bp DNA ladder was used as a size marker. Gels were visualized under UV with UVP GelStudio PLUS. The DNA banding patterns (i.e., DNA fingerprints) were analyzed using GelJ v2.3 software [21], and dendrograms were produced using Dice’s similarity coefficient (SD) with 0.5% position tolerance and the unweighted pair group method with arithmetic averages (UPGMA).

### Library preparation and sequencing

A representative strain for each of the unique PCR fingerprint types identified was randomly selected for whole genome sequencing. DNA from these 72 strains was normalized to 300 ng and prepared as individual libraries with the NEBNext Ultra II FS DNA Library Prep Kit. Each sample was uniquely indexed using NEBNext Multiplex Oligos for Illumina, and final libraries were cleaned with AMPure XP magnetic beads, quantified using the Qubit dsDNA HS Assay Kit, and profiled with an Agilent 2100 Bioanalyzer. Libraries were pooled and sequenced as paired-end 150 bp reads on a Novaseq System (Illumina; NovoGene).

### Quality filtering and sequence analysis

Raw sequence reads were quality filtered and trimmed with fastp [22] using default parameters with the flags -l 50 and -h. Snippy [23] was then implemented to call single nucleotide variants (SNVs) on the filtered and trimmed reads using the recently assembled genome of *P. mandapamensis* strain *Ik*.8.2 [12] as the reference, requiring a minimum coverage of 40x (--mincov 40) and minimum fraction of samples to be 90% (--minfrac 0.9). IQ-TREE [24] was then run on the full alignment of the genomes produced by snippy-core using the best predicted model with the lowest BIC score (TPM3 + F + R6) with up to 5000 bootstrap replicates. To characterize their variant effects, snpEff [25] was run on the set of SNVs identified by Snippy.

### Whole genome assembly

The genome for each strain was assembled with SPAdes [26] and subsequently scaffolded with RagTag [27] using the fully circularized genome of *P. mandapamensis* strain *Ik*.8.2 [12] as the reference. Sequences less than 1000 bp were discarded from the scaffolded assemblies to produce the final draft genomes for all downstream analyses. To assess genome completeness, BUSCO [28] was implemented using the Vibrionales (vibrionales_odb10) set of genes (*n* = 1445), and genome statistics were calculated with QUAST [21, 29, 30]. The assembled genomes were then annotated with Prokka [31].

FastANI was implemented on the complete set of genomes to quantify the average nucleotide identities (ANI) between strains. Several reference assemblies were also included in the ANI analysis for comparison, including the recently sequenced strain *Ik*.8.2 [12], the *P. mandapamensis* reference strain *svers*.1.1 (GCA_000211495.1) and *P. leiognathi* strain *lrivu*.4.1 (GCA_000509205.1). The results were visualized with ANIclustermap [32, 33].

### Pangenome and phylogenetic analysis

The pangenome of the unique symbiont strains was characterized using the Prokka annotations in Roary [34]. A phylogenetic analysis based on an alignment of the core genes was then carried out with IQ-TREE[24] using the best predicted model based on BIC scores (GTR + F + I + G4) and 2000 bootstrap replicates. The resulting phylogeny was then compared to the output tree based on the core alignment produced by Snippy. An additional phylogeny was inferred using the core gene alignment produced by Roary but including additional *Photobacterium* strains from NCBI as references and outgroups. The tree was constructed with IQ-TREE [24] using the same substitution model and 1200 bootstrap replicates.

### Growth and luminescence assays

Growth curves for strains Ph. A, Ph. C, Ph. D, Ph. V, Ph.EE, Ph.FF, Ph.GG, Ph.HH, and the reference strain *SV*.1.1 (GCA_030685315.1 [12]), were generated using a Biotek Synergy H1 Hybrid Multi-Mode Microplate Reader and the accompanying BioTek Gen5 Microplate Reader and Imager software (v3.10). Each strain was first grown on LSW-70 agar plates for 24 h at 26 °C. A single colony was then suspended in 3 mL of liquid LSW-70 media. After vortexing, 100 μL of each culture was added to a clear, flat bottom 96-well plate. The prepared 96-well plate was then put into the Biotek Synergy H1 Hybrid Multi-Mode Microplate Reader. Absorbance measurements were taken at 600 nm every 5 min for 24 h and recorded using the BioTek Gen5 Microplate Reader and Imager software (v3.10). All measurements were taken at room temperature with the 96-well plate lid covering the plate to reduce evaporation. Growth curves were analyzed in R (v4.2.1; R [35]) using the growthcurver package [36]. An ANOVA and TukeyHSD tests were performed to compare the intrinsic growth rate, r, between each strain.

Bioluminescence was measured for the same strains for which growth was measured (Ph. A, Ph. C, Ph. D, Ph. V, Ph.EE, Ph.FF, Ph.GG, Ph.HH, and SV.1.1) as well as for another non-luminous species of *Photobacterium*, *P. indicum* (ATCC 19614 ^T^) for comparison. Overnight cultures of each strain were grown at 26 °C in a New Brunswick Innova 43/43 R Console Incubator Shaker (Eppendorf) at 120 rpm and standardized to an OD of 0.9. Three mL of LSW-70 broth (*n* = 8 for each strain) was then inoculated with 100 uL of the overnight culture and incubated in a shaking incubator at 120 rpm and 26 °C for 24 h. The OD600 for each 3 mL culture was measured using a Biotek Synergy H1 Hybrid Multi-Mode Microplate Reader and standardized to an OD of 0.7 nm. The bioluminescence of a one mL aliquot from each 3 mL culture (*n* = 8) for each strain was measured using a GloMax 20/20 Luminometer (Promega) in a 1.5 mL microfuge tube. An ANOVA and TukeyHSD tests were performed to compare light production, measured as relative light units (RLU), between each strain in R (v4.2.1; R [35]).

## Results

### Strain fingerprinting reveals unique symbiont communities between individuals and locations

**Fig. 2:**
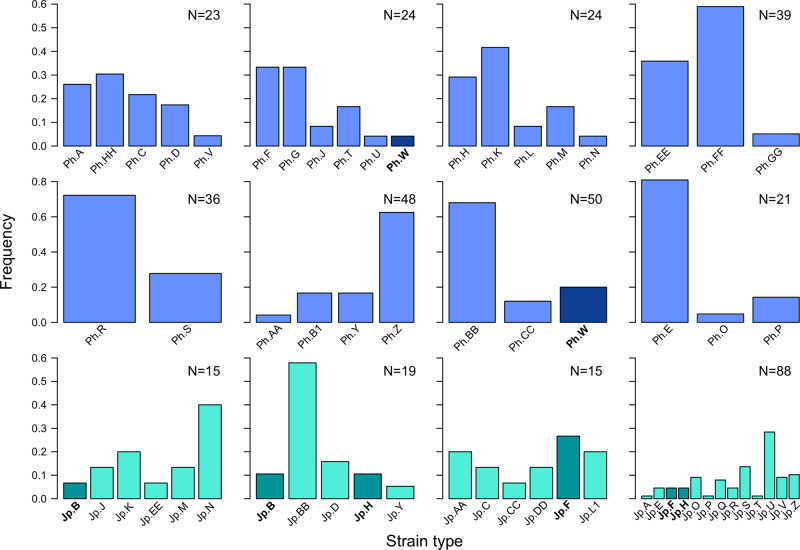
Relative abundances of uniquely identified strains of *Photobacterium mandapamensis* within the light organs of different *Siphamia tubifer* hosts identified by PCR fingerprinting. Each plot represents a different host. Blue plots represent hosts originating from the Philippines and green plots represent hosts from Japan. Dark bars indicate strains that were identified in more than one host.

ERIC-PCR fingerprinting of the symbiont isolates revealed unique patterns of distinct symbiont strain types present in all of the light organs examined (Figs. S1–S3). The number of strains identified within an individual host ranged from 1–13, with an average of six strains within a light organ. The relative abundances of the different strains also varied between individual hosts, with some hosts having a more even distribution of strains and others having a more skewed pattern (Fig. 2). One fish from the Philippines had only a single strain type identified from all 40 isolates and was therefore not included in Fig. 2. No strains isolated from either Japan or the Philippines were found in fish from the other location. There was also very little overlap in strains between individual hosts within each location; only a single strain was shared by two fish from the Philippines, and three strains were each shared by two fish from Japan (Fig. 2). One fish from Japan had a higher number of strains (*N* = 13) than all of the other fish examined, although this individual also had the highest number of isolates examined (*N* = 88). To determine whether sampling more isolates increases the observed strain diversity, we examined an additional 25 light organs and genotyped between 35–60 isolates from each using the same PCR-fingerprinting approach (Fig. S4). There was no correlation between the number of isolates screened and the number of strains detected (*R*^2^_adj_ = 0.057, *F*_1,36_ = 3.22, *p* = 0.081). There was also no significant correlation between fish standard length (a proxy for age) and the number strain types identified (*R*^2^_adj_ = 0.104, *F*_1,36_ = 5.31, *p* = 0.027; Fig. S5). The average number of strains detected within a light organ was 6.7 across all 38 hosts examined (Fig. S6).

### Genome assemblies of unique strain types

Representative strains for each of the unique PCR fingerprints identified were randomly selected for whole genome sequencing and assembly. Of these 72 strains, two were assembled to only three contigs, 28 strains had five or fewer contigs, and 62 contained ten or fewer contigs. The average depth of coverage for all of the genomes was 157x and ranged from 92–259x (Table S1). The average genome size was 4,800,573 bp, however, two genomes were considerably larger (>6.5 Mbp) than the others. The GC content averaged 40.98% across all genomes and the average number of coding sequences (CDS), rRNAs, and tRNAs were 4,150, 5, and 77, respectively. Nearly all of the genomes had BUSCO completeness scores greater than 99% for the Vibrionales (vibrionales_odb10) set of genes (*n* = 1445); the lowest BUSCO completeness score of all assemblies was 98.6% (Table S1).

### SNP and pangenome analysis

**Fig. 3:**
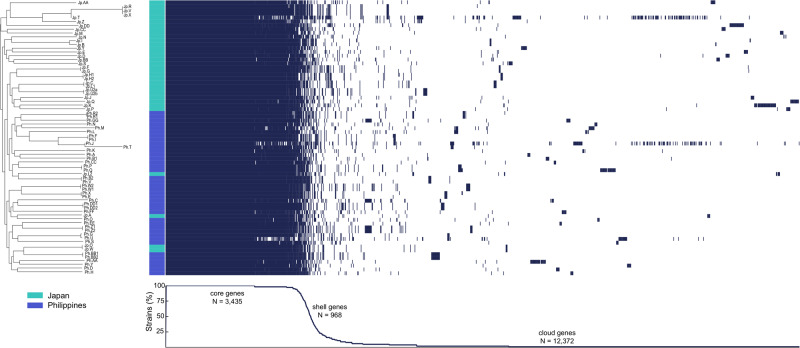
Pangenome analysis of the unique *Photobacterium mandapamensis* strains identified by PCR fingerprinting. The presence or absence of each gene is indicated by the dark blue color and the location of origin for each strain is specified by the corresponding color next to the plot. The number of core, shell, and cloud genes are shown on the bottom graph. The plot was generated with Phandango [51].

A total of 277,643 SNVs were identified across all 72 strains sequenced, 180,278 (73.7%) of which had synonymous effects. Of the remaining variants, 228 and 64,132 had nonsense and missense effects, respectively, for a missense to silent ratio of 0.36. The symbiont pangenome of was made up of 16,775 total genes, of which 3435 were core genes (present in 95–100% of the strains) with 2355 genes present in 99% or more of strains, 968 were shell genes (present in 15–95% of the strains), and 12,372 were “cloud” genes (present in fewer than 15% of the strains). Of the cloud genes identified, 7692 were singletons, present in only a single strain (Fig. 3). An analysis of the presence and absence of the *lux* genes indicated that all but two strains contained the complete operon, *luxCDABFE; luxF* was absent in two strains, Ph.C and Ph.FF, both originating from the Philippines.

### Phylogenetic analysis reveals location-specific symbiont clades

**Fig. 4:**
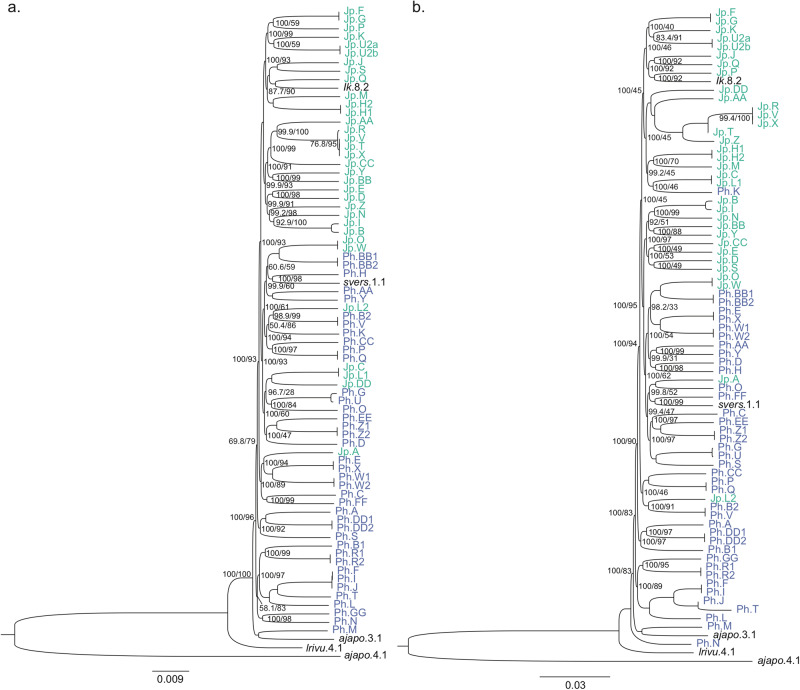
Phylogenetic relationships between the unique *Photobacterium mandapamensis* strains identified by PCR fingerprinting highlight location specific clades. Phylogenies were inferred (**a**) from the core genome alignment with 277,643 SNPs identified by Snippy [52] and (**b**) from an alignment of the 3435 core genes identified across all strains with Roary [37] using the GTR + F + I + G4 model. Tip labels are colored by their location of origin, either Japan (green) or Philippines (blue). Values listed at nodes represent the SH-aLRT/bootstrap scores. Nodes with no values listed had scores of 100/100.

A comparison of the inferred phylogenies based on the core set of 245,830 SNVs identified by Snippy and the core genome alignment of 3364 genes identified by Roary exhibit high overall congruency and indicated a clear distinction between the majority of strains from Japan and the Philippines (Fig. 4). All strains in both trees are placed among the *P. mandapamensis* reference strains, as opposed to *P. leiognathi*, suggesting they are all members of the *P. mandapamensis* subgroup. Additionally, there was support for a clade nested within both trees comprised of only strains from Japan, providing evidence of genetic divergence in the symbionts between the two locations. The most basal branches within the *P. mandapamensis* clade were primarily comprised of Philippine strains in both trees. One strain from the Philippines, Ph.M, was placed with *P. mandapamensis* strain *ajapo*.3.1 (isolated from the light organ of *Acropoma japonicum*, [10]) in both trees as opposed to belonging to the sister clade containing most of the remaining strains sequenced. The biggest discrepancy between these two phylogenies is the placement of strain Ph.N; in the SNV-based tree, Ph.N is placed in the basal Philippine clade, whereas in the core genome-based tree, Ph.N is placed as sister to all of the other *P. mandapamensis* strains but not grouped with the *P. leiognathi* reference strain, *lrivu*.4.1 (Fig. 4).

### Strain differences in bioluminescence and growth

**Fig. 5:**
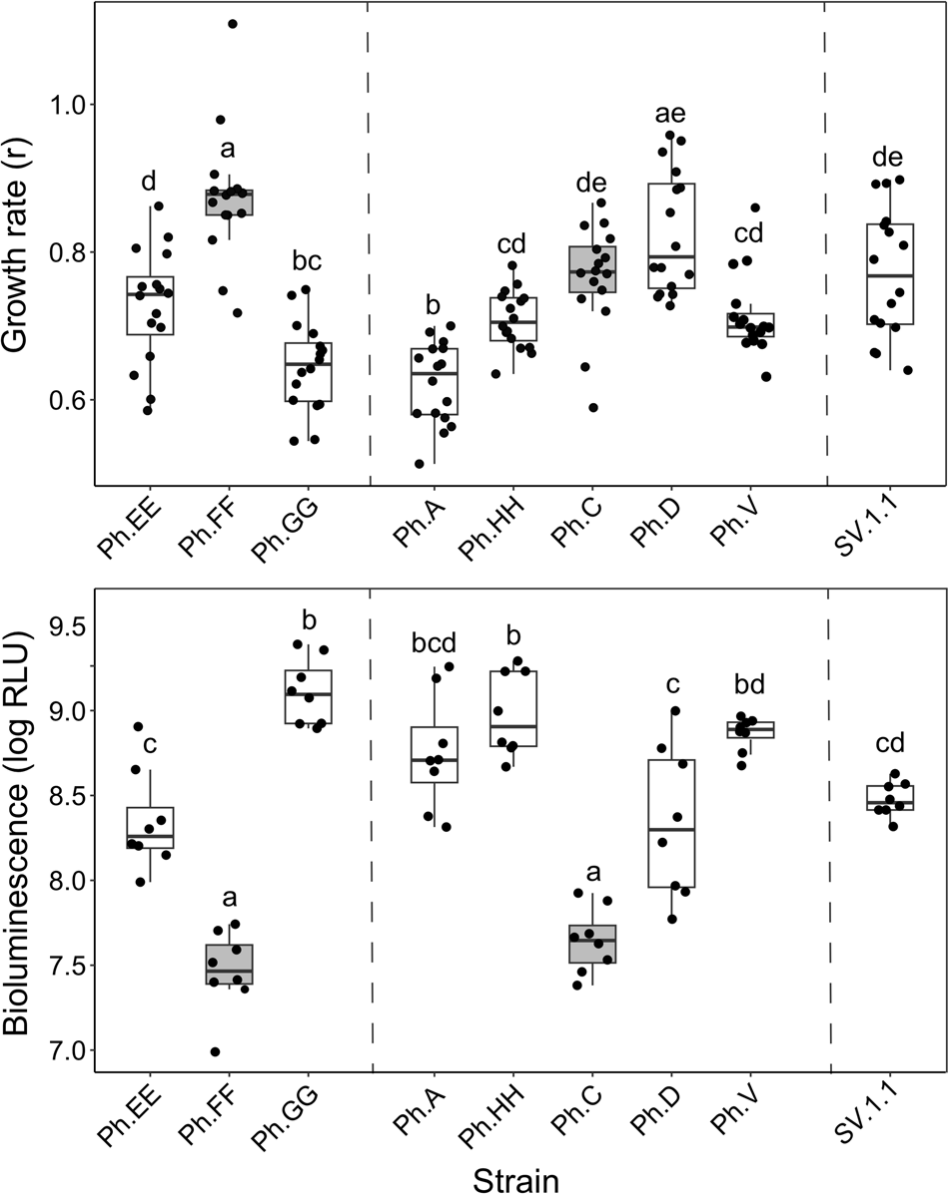
Strain variation in growth rates and bioluminescence of *Photobacterium mandapamensis* isolated from *Siphamia tubifer* light organs. Bioluminescence is shown as relative light units (RLU). Dashed lines depict distinctions between strains belonging to distinct light organ communities. Strains Ph.FF and Ph.C (gray) lack the *luxF* gene. Reference strain *SV*.1.1 (GCA_030685315.1) is included for comparison to the strains isolated in this study.

We measured the growth rates and luminosities of strains isolated from two light organs, one of which contained three strains and the other, five. One strain from each light organ community was missing the *luxF* gene (Ph.FF and Ph.C). Growth and luminosity of *P. mandapamensis* reference strain *SV*.1.1 (GCA_030685315.1 [12],) was also measured for comparison. Strains lacking *luxF* produced significantly less light than those with *luxF* (Fig. 5, Table S2). Overall, strain Ph.GG emitted the most light, averaging 1.4 × 10^9^ RLUs, and Ph.FF produced the least light, averaging 3.2 × 10^7^ RLUs. The other strain missing *luxF*, Ph.C, was the second most dim averaging 4.8 × 10^7^ RLUs (Fig. 5). The reference strain, *SV*.1.1, which contains *luxF*, emitted an average of 3.0 × 10^8^ RLUs. For comparison, the average light emission for the non-luminous strain, *P. indicum* (ATCC 19614 ^T^) was only 130 RLUs. Among the strains that contain *luxF* there were significant differences in light production (Table S2). For example, Ph.GG was significantly brighter than Ph. EE (*p.adj*. = 6.4e−06). Overall, there was a negative correlation between growth rate and luminosity; strains with higher light production had lower growth rates (*adj.R*^2^ = 0.5098, *p* = 0.019) (Fig. S7). In the three-member community, strains Ph.EE and Ph.GG, which produced significantly more light than Ph.FF, both had significantly lower growth rates (*p.adj*. = 9.34e−07 and 9.89e−14, respectively) (Fig. 5). The dimmest strain, Ph.FF, had the highest average growth rate (*r* = 0.87) compared to all other strains. Strain Ph.A had the lowest average growth rate (*r* = 0.62) and emitted an average of 7.4 × 10^8^ RLUs (Fig. 5). Within the five-member community, strain Ph.D had a the highest growth rate with an average r of 0.82, but was not significantly different than strain Ph.C, which lacks *luxF* (Fig. 5, Table S2).

## Discussion

Despite the high degree of specificity of the *S. tubifer*-*P. mandapamensis* symbiosis [9, 10], we observed significant strain-level differences in the symbiont communities between both host populations and individuals within a population. Genetic structure in the symbionts of *S. tubifer* was previously observed at spatial scales of only tens of kilometers despite a lack of structure in the host fish [16]. These results support the hypothesis that *S. tubifer* acquire their symbionts near their settlement site from the surrounding seawater, which is likely enriched with the symbiont genotypes from the established population of *S. tubifer* at that site [16]. Despite the structure between symbiont communities from different locations, there was little overlap in the symbiont strains observed between individuals within a population. This could be a result of the timing at which an individual fish arrives at its settlement site and which *P. mandapamensis* genotypes are enriched in the water at that time.

This study also confirms previous estimates that an average of six distinct symbiont genotypes occupy an individual *S. tubifer* light organ [16], although as seen here, this number can vary from as few as one to up to 13 distinct genotypes. Importantly, the number of strains does not increase with fish age, suggesting that the strain variation observed is not acquired throughout the host’s lifespan but is likely a result of the initial number of strains that colonize the light organ. Additionally, the relative abundance of each strain within a host varies between strains and individuals. For example, some symbiont communities are more even with respect to the relative abundances of individual strains, whereas other symbiont communities had one or two strains that were much more dominant. In the case of symbiont strains that were shared across fish hosts, they showed no similarity in terms of relative abundance within a host. For example, one strain (Jp.FF) that was most abundant in one light organ community had relatively low abundance in another (Fig. 2). This variability in strain dominance is also common in other microbial symbioses [37], including the human gut microbiome, in which a single strain is typically highly dominant within a species but the identity of the dominant strain can vary between individuals [38–40].

The ecological processes that result in distinct, strain-level symbiont communities between individual *S. tubifer* hosts remain unknown and require further investigation. For example, do some symbiont genotypes exhibit higher abundance within a light organ because they are better competitors in the host environment, or is their relative abundance within a host a reflection of colonization order and their availability in the seawater? Additionally, phenotypic differences between strains could have an effect on the composition of the final symbiont population that colonizes and persists within a host. *Allivibrio fischeri* strains that colonize squid light organs have different aggregation efficiencies, reach the crypts in the light organ at different speeds, and deploy their type VI secretion systems differently, which can affect their effectiveness at colonizing a host [41]. Similar phenotypic differences between *P. mandapamensis* strains could influence the community composition within *S. tubifer* light organs, although the type VI secretion system is not present in *P. mandapamensis*, and cause the observed differences in symbiont communities between individual hosts.

Strain-level differences in microbial symbionts are known to have critical fitness effects in other mutualistic host-microbe associations, including leguminous plants and their nitrogen-fixing *Rhizobia* symbionts [42] as well as for *Stienernema* nematodes and symbiotic *Xenorhabdus* bacteria [4]. Functional consequences for a host due to symbiont strain variation have also been documented in the honeybee gut microbiome, where the presence of distinct strains results in different abundances of various metabolites, altering the available nutrients for the host [43]. For bioluminescent symbiosis, the most apparent fitness effect for the host is light production by its bacterial symbiont, which is regulated by the *lux* operon [7]. In the case of the squid-vibrio association, *lux* mutant strains that produced less light inside a host squid were not able to colonize the light organ as effectively compared to wild-type strains [44]. Additionally, swelling of the light organ crypt cells, a critical step in host tissue differentiation, did not occur in the presence of *lux* mutants, indicating that bioluminescence is also essential for bacteria-host cell interaction in that system [45–47]. The effects of *lux* mutants and different symbiont genotypes on the *S. tubifer*-*P, mandapamensis* symbiosis have yet to be examined but could provide insight into the consequences of the diversity observed in the light organ symbiont communities between individual *S. tubifer* hosts.

The *lux* operon varies across different species of *Photobacterium* [7]. In *P. mandapamensis*, the *lux* operon is thought to be comprised of the *luxCDABFEG* genes, whereas the closely related parent species, *P. leiognathi*, lacks *luxF* [7]. A recent study examining the genomes of *Photobacterium* spp. isolated from the light organs of various fish species revealed several *P. mandapamensis* strains lacking *luxF*, although all strains isolated from *S. tubifer* hosts contained the gene [12]. However, in this study, two strains isolated from *S. tubifer* lacked *luxF*, indicating that this gene is not required for the bioluminescent symbiosis with *Siphamia* hosts but could still play a role in the association*. LuxF* has been shown to increase light emission when cloned into *E. coli* [48], but its role in light production in *P. mandapamensis* has not been described. We show that strains Ph.FF and Ph.C, both of which lack *luxF*, produced significantly less light than strains isolated from the same light organs that contain *luxF*. There was also a significant negative correlation between light production and growth, suggesting that increased light production could come with a metabolic cost for the bacteria. Therefore, strains lacking *luxF* could have a growth advantage inside the host environment. Strain Ph.FF was also the most abundant of the three strains present in light organ community, providing evidence that this potential trade-off could potentially provide a competitive advantage for strains producing less light within the host. Additionally, light production and growth rates varied between many of the strains containing *luxF* indicating there are other physiological differences between strains and highlighting the need to consider intraspecific variability in microbial symbionts more closely. More detailed studies of the processes regulating the symbiont community structure within a light organ and the consequences of this variation, specifically with respect to symbiont growth and metabolism and the potential consequences for the host, will help reveal the impacts of strain-level variation on the *S. tubifer*-*P. mandapamensis* symbiosis.

Strain specificity in microbial symbiosis can have critical impacts on the association, yet this level of symbiont diversity is commonly overlooked and poorly defined for most systems. We observed considerable sub-species, strain-level diversity in an otherwise highly specific, binary association between a marine fish and a luminous bacterium. However, whether the host fish has any control over which symbiont strains can colonize and/or persist within its light organ or if the symbiont communities observed are purely a reflection of strain colonization order and competition remains unknown. We also identified distinct symbiont lineages specific to the two locations examined in this study. This could be due to ecological processes that influence which symbiont genotypes are available to larval fish hosts in each location during the window of colonization or alternatively, it could be a reflection of local host preference for native symbiont strains. Studies of the luminous symbionts of sepiolid squids over fairly large geographic scales showed a competitive advantage of native symbiont strains in colonizing *E. scolopes* light organs [49, 50]. Future studies determining whether *S. tubifer* hosts can discriminate between local and “foreign” symbionts would provide more insight into the processes structuring these geographically distinct symbiont communities. The high amount of strain-level diversity observed for the otherwise highly specific bioluminescent *S. tubifer-P. mandapamensis* symbiosis provides an ideal opportunity to investigate the functional consequences of strain diversity for the host and the symbiosis as well as the underlying ecological and physiological processes involved in structuring symbiont communities.

### Supplementary information

Supplemental Material

## Data Availability

Sequence data for this study are accessible in the GenBank database under the project number PRJNA1028546 (SAMN37845189-SAMN37845260).
