# Supplementary material for: Strain-level diversity of symbiont communities between individuals and populations of a bioluminescent fish
Source: ISME J. 2023 Oct 27;17(12):2362–9. doi: 10.1038/s41396-023-01550-6 (PMC10689835; doi:10.1038/s41396-023-01550-6)
Supplement: Supplementary file 1 — Supplemental Material [file 41396_2023_1550_MOESM1_ESM.pdf]

Summary

This document contains supplemental figures and tables for the manuscript: “Strain-level diversity of symbiont communities between individuals and populations of a bioluminescent fish host” by AL Gould, SA Donohoo, ED Román, and E Neff

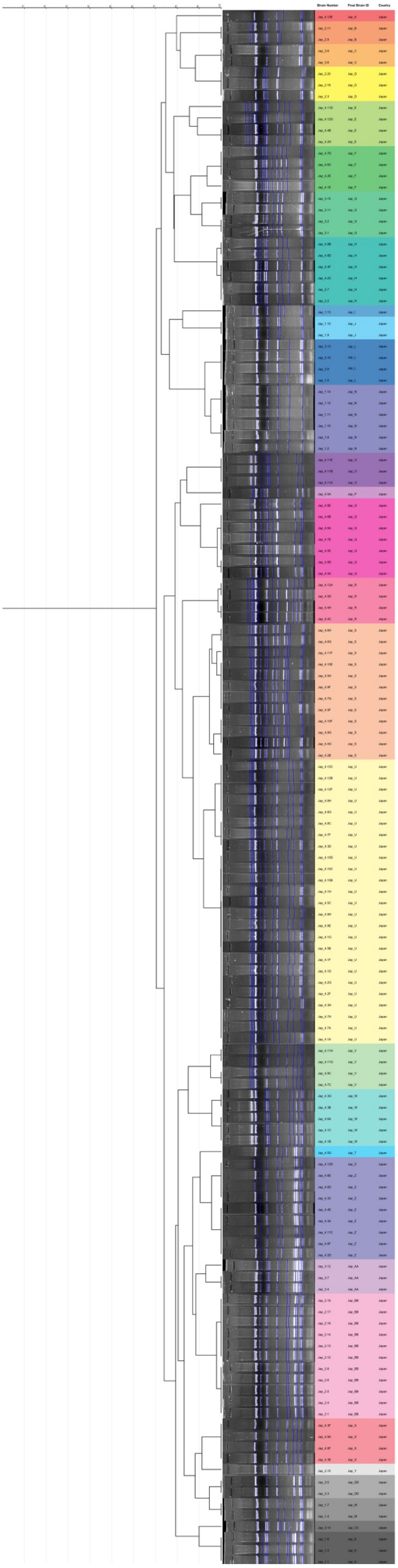

Figure S1. Gel images of the ERIC-PCR products depicting the banding patterns (or "fingerprints") of the *Photobacterium mandapamensis* strains isolated from *Siphamia tubifer* light organs from Japan. Gel images were processed with GelJ software.

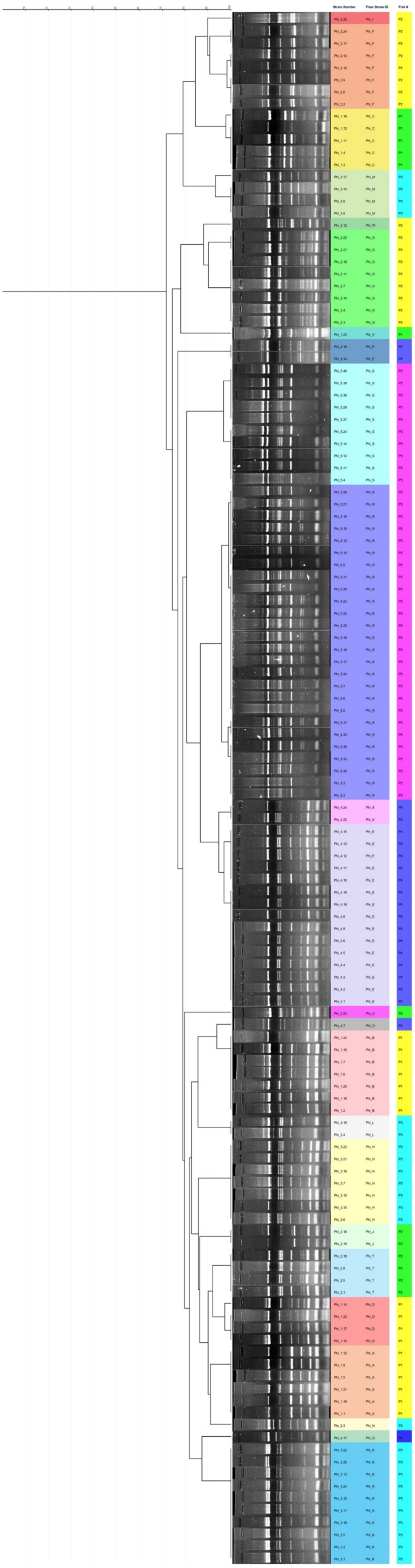

Figure S2. Gel images of the ERIC-PCR products depicting the banding patterns (or "fingerprints") of the *Photobacterium mandapamensis* strains isolated from *Siphamia tubifer* light organs from Philippines. Gel images were processed with GelJ software.

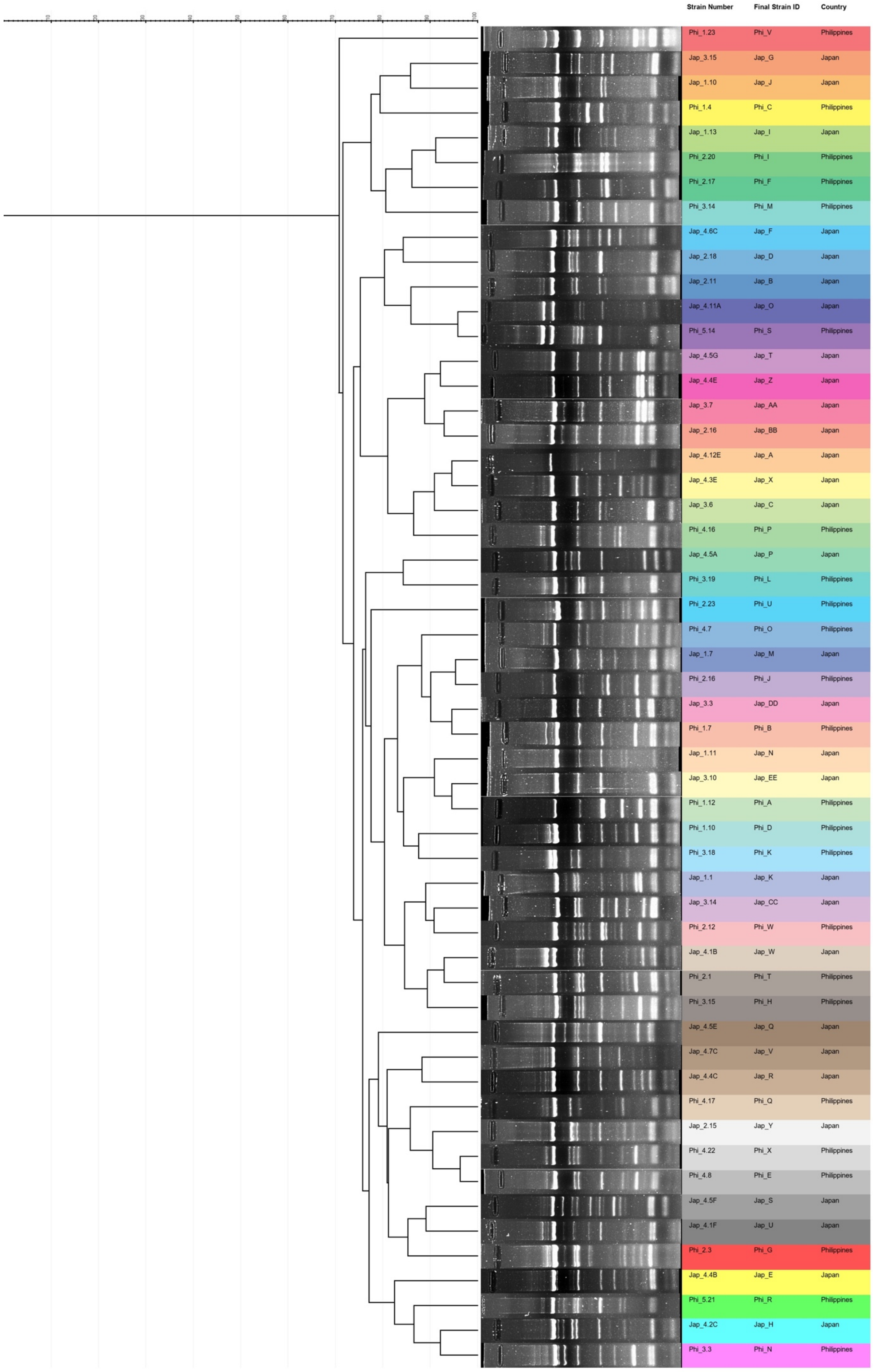

Figure S3. Gel images of the ERIC-PCR products depicting the banding patterns (or "fingerprints") of the unique *Photobacterium mandapamensis* strains isolated from *Siphamia tubifer* light organs from Japan and the Philippines. Gel images were processed with GelJ software.

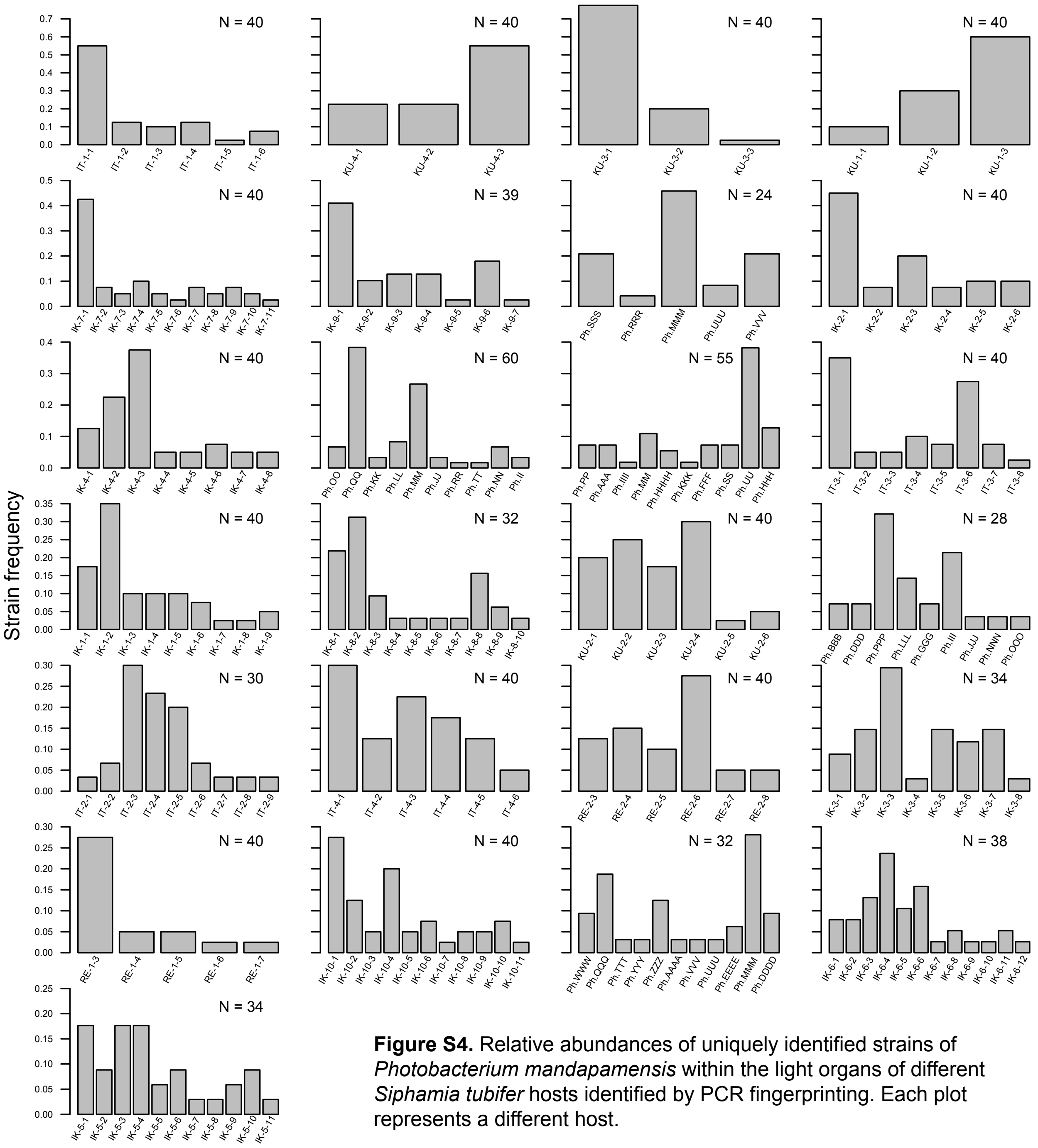

Figure S4. Relative abundances of uniquely identified strains of *Photobacterium mandapamensis* within the light organs of different *Siphamia tubifer* hosts identified by PCR fingerprinting. Each plot represents a different host.

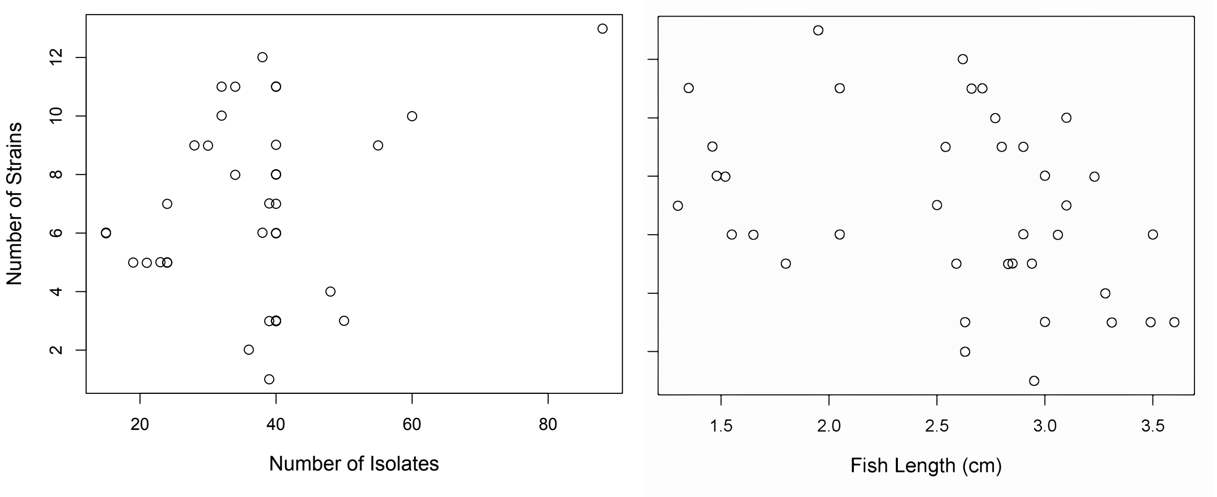

Figure S5. The number of unique strains identified by ERIC-PCR fingerprinting within an individual *Siphamia tubifer* light organ versus the number of isolates that were screened (left) and the standard length of the fish host (right).

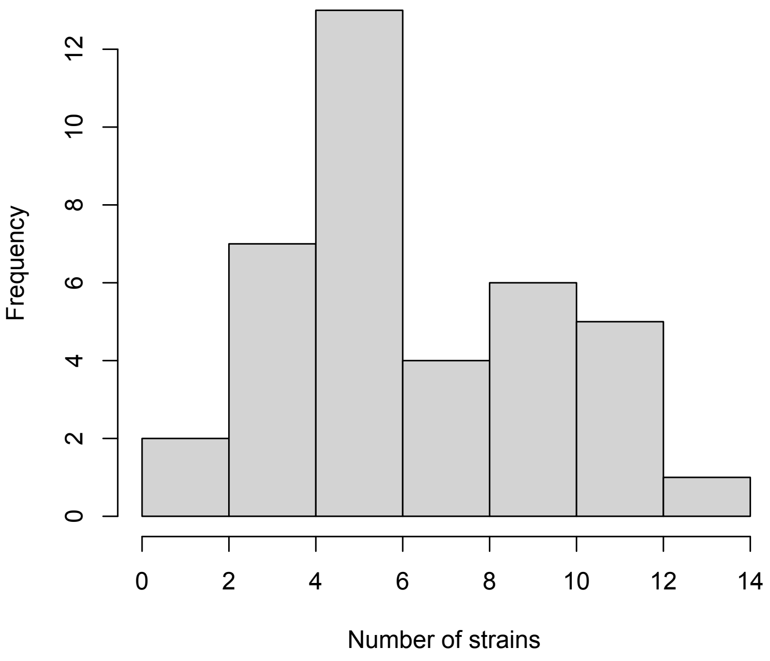

Figure S6. The frequency distribution of the number of distinct strain types identified by ERIC-PCR within an individual *Siphamia tubifer* light organ (n = 38).

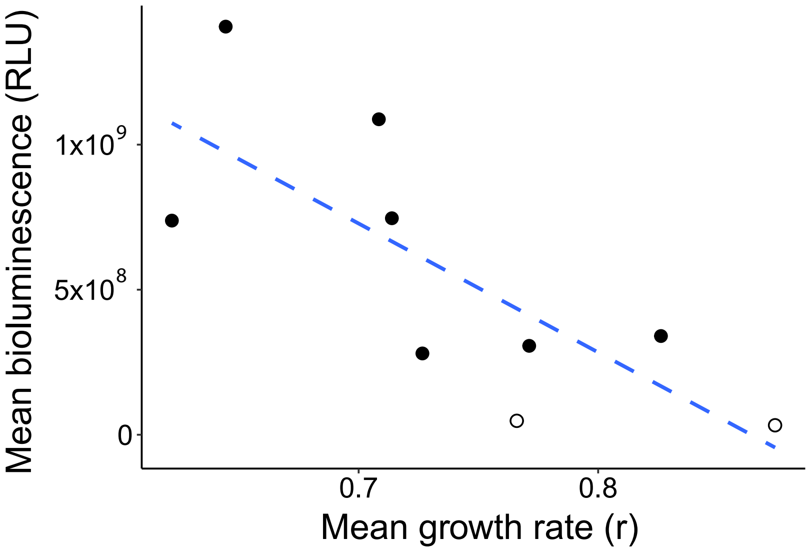

Figure S7. The mean bioluminescence of each strain examined in this study as a function of its mean growth rate (n=8 for each strain). Open circles indicate strains lacking *luxF*. There is a negative correlation between light emission and growth (*adj.*R^2= 0.5098, p = 0.019)

Table S1. Summary statistics of the genome assemblies of the *Photobacterium mandapamensis* strains isolated from *Siphamia tubifer* light organs and sequenced in this study. BUSCO scores listed are the percentage of the Vibrionales set of orthologs that are complete (C) as single copies (S) or duplicates (D), fragmented (F), or missing (M).

strain ID	raw reads	trimmed reads	coverage	contigs	total bp	largest contig	%GC	N50	L50	#Ns/100 kbp	CDS	rRNA	tRNA	tmRNA	repreat	BUSCO
Jp.A	4527508	4478356	145	4	4633672	3073759	41.06	3073759	1	103.59	4074	4	82	1	0	C:99.2 [S:98.8 D:0.4] F:0.1 M:0.7
Jp.AA	7885318	7802690	249	5	4696766	3106696	40.99	3106696	1	78.78	4079	6	73	1	0	C:99.1 [S:98.8 D:0.3] F:0.1 M:0.8
Jp.B	5244200	5180042	166	9	4670715	3073125	41.03	3073125	1	128.46	4080	6	88	1	0	C:99.1 [S:98.8 D:0.3] F:0.1 M:0.8
Jp.BB	4758844	4703520	148	5	4754222	3162411	40.96	3162411	1	90.45	4200	5	78	1	0	C:99.2 [S:98.8 D:0.4] F:0.1 M:0.7
Jp.C	5216142	5161904	163	5	4745841	3133782	41	3133782	1	139.07	4159	5	79	1	0	C:98.9 [S:98.6 D:0.3] F:0.1 M:1.0
Jp.CC	4085318	4012058	126	10	4780606	3086804	40.95	3086804	1	106.68	4203	4	72	1	0	C:99.1 [S:98.7 D:0.4] F:0.1 M:0.8
Jp.D	4420742	5556712	166	11	5031723	3141418	40.74	3141418	1	95.39	4505	8	79	1	0	C:99.1 [S:98.8 D:0.3] F:0.1 M:0.8
Jp.DD	5687770	4382888	142	5	4627685	3076312	40.97	3076312	1	95.08	4062	3	73	1	0	C:99.0 [S:98.6 D:0.4] F:0.1 M:0.9
Jp.E	3923718	3847700	124	6	4656775	3081565	41.02	3081565	1	120.25	4090	5	64	1	1	C:99.2 [S:98.8 D:0.4] F:0.1 M:0.7
Jp.F	6136302	6065970	189	8	4808101	3143585	40.94	3143585	1	101.91	4224	5	73	1	0	C:99.1 [S:98.8 D:0.3] F:0.1 M:0.8
Jp.G	5235402	5056690	158	7	4802553	3141378	40.95	3141378	1	135.34	4227	5	68	1	0	C:99.1 [S:98.8 D:0.3] F:0.1 M:0.8
Jp.H1	3994338	3947794	124	9	4770762	3126342	40.97	3126342	1	119.48	4182	4	73	1	1	C:98.9 [S:98.6 D:0.3] F:0.1 M:1.0
Jp.H2	5622376	5561770	175	8	4768750	3123910	40.97	3123910	1	113.24	4180	4	73	1	1	C:99.0 [S:98.7 D:0.3] F:0.1 M:0.9
Jp.I	4300370	4242600	135	12	4706057	3072444	40.96	3072444	1	125.37	4092	6	79	1	0	C:99.1 [S:98.8 D:0.3] F:0.1 M:0.8
Jp.J	4508106	4421288	139	9	4757494	3081725	40.98	3081725	1	113.51	4179	6	83	1	0	C:99.1 [S:98.8 D:0.3] F:0.1 M:0.8
Jp.K	5389504	5309854	150	13	5307567	3105730	40.4	3105730	1	94.21	4743	6	74	1	0	C:99.1 [S:98.7 D:0.4] F:0.1 M:0.8
Jp.L1	3750378	3709080	116	9	4780741	3088785	40.95	3088785	1	123.41	4199	6	77	1	0	C:98.9 [S:98.6 D:0.3] F:0.1 M:1.0
Jp.L2	5582496	5495892	173	8	4764203	3065282	41	3065282	1	96.55	4187	6	75	1	1	C:99.1 [S:98.8 D:0.3] F:0.1 M:0.8
Jp.M	6673512	6576300	214	3	4616365	3079317	41.02	3079317	1	115.03	4046	6	73	1	0	C:99.0 [S:98.7 D:0.3] F:0.1 M:0.9
Jp.N	4199692	4156578	135	4	4628744	3091952	41	3091952	1	129.62	4058	3	71	1	0	C:99.0 [S:98.7 D:0.3] F:0.1 M:0.9
Jp.O	5875214	5808314	188	4	4627800	3069180	41.05	3069180	1	114.53	4038	5	80	1	2	C:99.1 [S:98.8 D:0.3] F:0.1 M:0.8
Jp.P	5710306	5640692	179	9	4724157	3094261	40.94	3094261	1	118.54	4130	5	73	1	0	C:99.2 [S:98.8 D:0.4] F:0.1 M:0.7
Jp.Q	3738754	3689164	114	10	4833662	3135250	40.96	3135250	1	115.85	4248	7	71	1	1	C:99.2 [S:98.8 D:0.4] F:0.1 M:0.7
Jp.R	4117700	4071822	129	9	4731975	3080104	40.99	3080104	1	109.89	4164	6	87	1	0	C:99.1 [S:98.8 D:0.3] F:0.1 M:0.8
Jp.S	4591496	4509522	145	7	4664477	3094385	40.97	3094385	1	126.49	4083	4	73	1	0	C:99.2 [S:98.9 D:0.3] F:0.1 M:0.7

Jp.T	5453120	5377364	109	486	7412758	3521377	40.93	1751682	2	3386.05	5955	4	75	1	0	C:98.8 [S:76.0 D:22.8] F:0.1 M:1.1
Jp.U2a	5081690	5023074	160	8	4723587	3135267	40.95	3135267	1	116.44	4137	5	73	1	0	C:98.8 [S:98.5 D:0.3] F:0.1 M:1.1
Jp.U2b	6787788	6734826	214	8	4724154	3136146	40.95	3136146	1	116.42	4135	5	75	1	0	C:98.8 [S:98.5 D:0.3] F:0.1 M:1.1
Jp.V	3461826	3417866	108	9	4725332	3080928	41	3080928	1	95.23	4155	6	84	1	0	C:99.1 [S:98.8 D:0.3] F:0.1 M:0.8
Jp.W	6059482	5988822	194	5	4628115	3068370	41.05	3068370	1	116.68	4040	5	73	1	2	C:99.1 [S:98.8 D:0.3] F:0.1 M:0.8
Jp.X	4048144	3967582	126	8	4726491	3083500	41	3083500	1	107.9	4155	6	89	1	1	C:99.1 [S:98.8 D:0.3] F:0.1 M:0.8
Jp.Y	4206188	4164178	134	5	4677896	3117401	40.98	3117401	1	98.33	4118	6	73	1	0	C:99.1 [S:98.8 D:0.3] F:0.1 M:0.8
Jp.Z	6121994	6064872	196	4	4640308	3094643	40.95	3094643	1	133.61	4056	5	81	1	0	C:99.0 [S:98.7 D:0.3] F:0.1 M:0.9
Ph.A	3782586	3732798	120	5	4654544	3102581	41.02	3102581	1	124.61	4045	4	74	1	1	C:99.2 [S:98.9 D:0.3] F:0.1 M:0.7
Ph.AA	7409938	7311774	222	7	4938808	3126932	40.86	3126932	1	113.39	4370	5	82	1	0	C:99.1 [S:98.8 D:0.3] F:0.1 M:0.8
Ph.B1	6662510	6556676	214	4	4589320	3087633	41.08	3087633	1	89.34	4016	3	79	1	0	C:99.2 [S:98.8 D:0.4] F:0.1 M:0.7
Ph.HH	2990816	2954300	96	4	4597598	2887726	41.05	2887726	1	121.8	4015	4	73	1	0	C:99.1 [S:98.8 D:0.3] F:0.1 M:0.8
Ph.BB1	4045402	3983630	124	12	4804694	3143962	40.98	3143962	1	116.55	4233	4	84	1	1	C:99.2 [S:98.8 D:0.4] F:0.1 M:0.7
Ph.BB2	7226018	7131760	223	13	4803734	3144816	40.98	3144816	1	108.25	4228	4	80	1	2	C:99.2 [S:98.8 D:0.4] F:0.1 M:0.7
Ph.C	4337528	4229108	139	4	4573587	3076013	41.07	3076013	1	91.83	4010	5	85	1	0	C:98.9 [S:98.5 D:0.4] F:0.1 M:1.0
Ph.CC	5773410	5695462	177	4	4820728	2938017	40.94	2938017	1	134.83	4294	3	74	1	1	C:99.2 [S:98.8 D:0.4] F:0.1 M:0.7
Ph.D	4022158	3290348	103	10	4803580	3211531	40.87	3211531	1	89.52	4180	4	75	1	0	C:99.1 [S:98.8 D:0.3] F:0.1 M:0.8
Ph.DD1	3330322	6616710	207	8	4805006	3182180	40.87	3182180	1	93.65	4180	4	89	1	0	C:99.1 [S:98.8 D:0.3] F:0.1 M:0.8
Ph.DD2	6715924	3965944	127	6	4687992	3073541	40.98	3073541	1	151.45	4127	5	66	1	0	C:99.1 [S:98.8 D:0.3] F:0.1 M:0.8
Ph.E	4650820	5396898	176	6	4587290	3037058	41.03	3037058	1	95.92	4008	6	82	1	0	C:99.1 [S:98.8 D:0.3] F:0.1 M:0.8
Ph.EE	5467404	4598816	150	5	4606939	3115225	41.02	3115225	1	115.04	4021	4	80	1	0	C:99.0 [S:98.7 D:0.3] F:0.1 M:0.9
Ph.F	4613102	8103342	259	7	4696900	3100670	40.99	3100670	1	100.07	4119	5	80	1	2	C:99.1 [S:98.8 D:0.3] F:0.1 M:0.8
Ph.FF	8220120	4531582	147	5	4633009	3088120	41	3088120	1	110.08	4040	4	86	1	0	C:99.1 [S:98.7 D:0.4] F:0.1 M:0.8
Ph.G	3352176	7344928	229	6	4821204	3099340	40.94	3099340	1	112.01	4273	4	74	1	1	C:98.9 [S:98.5 D:0.4] F:0.1 M:1.0
Ph.GG	7435050	3303946	107	5	4643646	3087658	41.01	3087658	1	94.75	4071	6	74	1	0	C:99.1 [S:98.8 D:0.3] F:0.1 M:0.8
Ph.H	4607086	4531814	147	7	4631348	3005076	41.02	3005076	1	127.39	4049	4	75	1	0	C:99.1 [S:98.7 D:0.4] F:0.1 M:0.8
Ph.I	4061420	4019640	130	4	4629003	3090932	41.01	3090932	1	103.69	4031	4	82	1	0	C:99.1 [S:98.8 D:0.3] F:0.1 M:0.8
Ph.J	6658300	6561456	145	384	6790031	3431672	40.97	3431672	1	3455.07	5489	3	89	1	0	C:98.6 [S:80.1 D:18.5] F:0.2 M:1.2
Ph.K	6499018	6407478	208	5	4619654	3076376	41.02	3076376	1	112.56	4033	6	76	1	0	C:99.2 [S:98.8 D:0.4] F:0.1 M:0.7

Ph.L	5187138	5120996	158	11	4851295	3085275	40.89	3085275	1	111.31	4236	5	73	1	3	C:99.2 [S:98.8 D:0.4] F:0.1 M:0.7
Ph.M	6514524	6444012	197	12	4905575	3140432	40.85	3140432	1	142.69	4289	6	81	1	0	C:99.1 [S:98.5 D:0.6] F:0.1 M:0.8
Ph.N	4080480	3990172	128	6	4676387	3106615	41.04	3106615	1	106.92	4071	5	81	1	0	C:99.1 [S:98.8 D:0.3] F:0.1 M:0.8
Ph.O	5835460	5761852	187	6	4611444	3063112	41.06	3063112	1	95.41	4045	3	73	1	0	C:99.1 [S:98.8 D:0.3] F:0.1 M:0.8
Ph.P	7049054	6966586	223	7	4682271	3080219	41	3080219	1	102.51	4091	3	76	1	0	C:99.2 [S:98.8 D:0.4] F:0.1 M:0.7
Ph.Q	3837624	3791986	110	10	5161199	3366649	41.44	3366649	1	100.75	4535	4	84	1	0	C:99.4 [S:84.8 D:14.6] F:0.1 M:0.5
Ph.R1	2973310	2932698	92	5	4792509	3147317	41	3147317	1	91.81	4195	3	74	1	1	C:99.2 [S:98.8 D:0.4] F:0.1 M:0.7
Ph.R2	4449070	4388676	137	7	4790181	3092047	41	3092047	1	93.94	4193	3	78	1	1	C:99.2 [S:98.8 D:0.4] F:0.1 M:0.7
Ph.S	5629422	5537748	179	3	4646439	3106535	40.98	3106535	1	88.24	4066	4	65	1	0	C:99.0 [S:98.7 D:0.3] F:0.1 M:0.9
Ph.T	4767326	4661778	150	5	4655466	3095283	41.01	3095283	1	92.36	4050	5	91	1	0	C:99.2 [S:98.8 D:0.4] F:0.1 M:0.7
Ph.U	3421824	3385214	102	93	4976817	3194402	41	3194402	1	3327.43	4176	2	64	1	0	C:91.9 [S:90.0 D:1.9] F:3.6 M:4.5
Ph.V	6023072	5956028	194	4	4603705	3118577	41.04	3118577	1	117.3	4021	4	70	1	0	C:99.1 [S:98.8 D:0.3] F:0.1 M:0.8
Ph.W1	5320454	5249668	165	9	4782911	3191443	40.92	3191443	1	98.27	4191	5	73	1	0	C:99.1 [S:98.8 D:0.3] F:0.1 M:0.8
Ph.W2	3923852	3860014	121	9	4779907	3188252	40.92	3188252	1	100.42	4188	5	75	1	0	C:99.1 [S:98.8 D:0.3] F:0.1 M:0.8
Ph.X	4796704	4744506	153	9	4660838	3113188	41.11	3113188	1	115.86	4054	6	79	1	0	C:99.2 [S:97.3 D:1.9] F:0.1 M:0.7
Ph.Y	5648888	5559262	179	5	4650016	3102968	40.99	3102968	1	107.53	4052	5	83	1	0	C:99.1 [S:98.8 D:0.3] F:0.1 M:0.8
Ph.Z1	3245874	3201216	101	5	4762254	3033823	40.96	3033823	1	75.59	4231	5	85	1	0	C:99.0 [S:98.7 D:0.3] F:0.1 M:0.9
Ph.Z2	5477586	5340984	168	5	4763345	3034353	40.96	3034353	1	92.37	4232	5	83	1	0	C:99.0 [S:98.7 D:0.3] F:0.1 M:0.9

Table S2. Results of Tukey's post-hoc tests for the analysis of variance (ANOVA) for the growth rates and bioluminescence of all pair-wise comparisons of the strains tested in this study.

Interaction Pairs	Growth rate (r)				Bioluminescence (log RLU)			
	diff	lwr	upr	p adj	diff	lwr	upr	p adj
**Ph.FF - Ph.EE**	0.1472	0.0687	0.2258	0.0000	-0.8820	-1.3004	-0.4636	0.0000
**Ph.GG - Ph.EE**	-0.0821	-0.1607	-0.0036	0.0330	0.7630	0.3446	1.1814	0.0000
**Ph.A - Ph.EE**	-0.1046	-0.1831	-0.0261	0.0016	0.4040	-0.0143	0.8224	0.0664
**Ph.HH - Ph.EE**	-0.0182	-0.0967	0.0603	0.9983	0.6296	0.2112	1.0479	0.0003
**Ph.C - Ph.EE**	0.0394	-0.0391	0.1179	0.8132	-0.7019	-1.1202	-0.2835	0.0000
**Ph.D - Ph.EE**	0.0996	0.0211	0.1781	0.0033	-0.0050	-0.4234	0.4133	1.0000
**Ph.V - Ph.EE**	-0.0128	-0.0913	0.0658	0.9999	0.5171	0.0987	0.9355	0.0055
**SV1.1 - Ph.EE**	0.0446	-0.0340	0.1231	0.6888	0.1297	-0.2887	0.5480	0.9850
**Ph.GG - Ph.FF**	-0.2294	-0.3079	-0.1508	0.0000	1.6450	1.2266	2.0634	0.0000
**Ph.A - Ph.FF**	-0.2518	-0.3304	-0.1733	0.0000	1.2860	0.8676	1.7044	0.0000
**Ph.HH - Ph.FF**	-0.1654	-0.2440	-0.0869	0.0000	1.5116	1.0932	1.9299	0.0000
**Ph.C - Ph.FF**	-0.1078	-0.1864	-0.0293	0.0009	0.1801	-0.2383	0.5985	0.9001
**Ph.D - Ph.FF**	-0.0476	-0.1262	0.0309	0.6062	0.8769	0.4586	1.2953	0.0000
**Ph.V - Ph.FF**	-0.1600	-0.2385	-0.0815	0.0000	1.3991	0.9807	1.8174	0.0000
**SV1.1 - Ph.FF**	-0.1027	-0.1812	-0.0241	0.0021	1.0116	0.5933	1.4300	0.0000
**Ph.A - Ph.GG**	-0.0225	-0.1010	0.0561	0.9925	-0.3590	-0.7773	0.0594	0.1498
**Ph.HH - Ph.GG**	0.0639	-0.0146	0.1425	0.2106	-0.1334	-0.5518	0.2850	0.9820
**Ph.C - Ph.GG**	0.1215	0.0430	0.2001	0.0001	-1.4649	-1.8832	-1.0465	0.0000
**Ph.D - Ph.GG**	0.1817	0.1032	0.2603	0.0000	-0.7680	-1.1864	-0.3497	0.0000
**Ph.V - Ph.GG**	0.0694	-0.0092	0.1479	0.1295	-0.2459	-0.6643	0.1725	0.6243
**SV1.1 - Ph.GG**	0.1267	0.0482	0.2052	0.0000	-0.6333	-1.0517	-0.2150	0.0003
**Ph.HH - Ph.A**	0.0864	0.0079	0.1649	0.0195	0.2255	-0.1928	0.6439	0.7251
**Ph.C - Ph.A**	0.1440	0.0655	0.2225	0.0000	-1.1059	-1.5243	-0.6875	0.0000
**Ph.D - Ph.A**	0.2042	0.1257	0.2827	0.0000	-0.4091	-0.8275	0.0093	0.0602

**Ph.V - Ph.A**	0.0919	0.0133	0.1704	0.0096	0.1130	-0.3053	0.5314	0.9939
**SV1.1 - Ph.A**	0.1492	0.0707	0.2277	0.0000	-0.2744	-0.6928	0.1440	0.4784
**Ph.C - Ph.HH**	0.0576	-0.0209	0.1361	0.3426	-1.3314	-1.7498	-0.9131	0.0000
**Ph.D - Ph.HH**	0.1178	0.0392	0.1963	0.0002	-0.6346	-1.0530	-0.2162	0.0003
**Ph.V - Ph.HH**	0.0054	-0.0731	0.0840	1.0000	-0.1125	-0.5309	0.3059	0.9941
**SV1.1 - Ph.HH**	0.0628	-0.0158	0.1413	0.2320	-0.4999	-0.9183	-0.0815	0.0083
**Ph.D - Ph.C**	0.0602	-0.0183	0.1387	0.2836	0.6968	0.2784	1.1152	0.0000
**Ph.V - Ph.C**	-0.0521	-0.1307	0.0264	0.4821	1.2189	0.8006	1.6373	0.0000
**SV1.1 - Ph.C**	0.0052	-0.0733	0.0837	1.0000	0.8315	0.4132	1.2499	0.0000
**Ph.V - Ph.D**	-0.1123	-0.1909	-0.0338	0.0005	0.5221	0.1037	0.9405	0.0049
**SV1.1 - Ph.D**	-0.0550	-0.1335	0.0235	0.4063	0.1347	-0.2837	0.5531	0.9809
**SV1.1 - Ph.V**	0.0573	-0.0212	0.1359	0.3486	-0.3874	-0.8058	0.0310	0.0909
